# Missed Opportunities in Maternal Care: A Cross-Sectional Study of Utilisation of Anganwadi Services in Central India

**DOI:** 10.7759/cureus.97869

**Published:** 2025-11-26

**Authors:** Amruta Choudhary, Anita Yadav, Rubina Bano Rangrej, Arun S Sanap, Mubashara Khan, Anusha Kamath, Neha N Gangane, Shuchita Mundle

**Affiliations:** 1 Department of Obstetrics and Gynaecology, All India Institute of Medical Sciences, Nagpur, Nagpur, IND; 2 Department of Community Medicine, All India Institute of Medical Sciences, Nagpur, Nagpur, IND

**Keywords:** aganwadi services, healthcare utilisation, icds, non utilisation, supplementary nutrition

## Abstract

Introduction

The Government of India has initiated several programs to enhance maternal health. Despite comprehensive operations and significant governmental backing, the usage of nutrition aid and healthcare services from Anganwadi Centres for pregnant and breastfeeding women remains inconsistent and insufficient in various locations.

Objectives

The primary objective was to investigate the factors influencing the utilization of these services and to explore the reasons for non-utilization.

Methodology

This was a cross-sectional observational study, intended to evaluate the utilization of Anganwadi services among pregnant and breastfeeding mothers in Central India. A total of 365 pregnant women and lactating mothers were interviewed using a face-validated questionnaire covering demographic characteristics, awareness of Anganwadi services, and factors influencing utilization.

Results

The study participants were maximum in the age range of 20 to 30 years, with 35.8% had done graduation. About 61.91% of participants had a spouse qualification of less than graduation. The utilization rate of Anganwadi services among the study participants was around 91%. However, challenges such as logistical barriers, concerns about service quality, and disparities in access persisted. Statistical analysis indicated a significant association between these factors and service utilization (p < 0.05).

Conclusion

The findings have highlighted the need for targeted interventions to ensure equitable access to maternal and child healthcare services and have significant implications for policy development and program planning.

## Introduction

Nutrition during pregnancy and lactation is crucial for the health and wellness of both the mother and baby. A mother's nutritional status during pregnancy not only influences her breast milk composition, her health in future pregnancies, but also influences the developmental and physiological metabolism of offspring, thus affecting the worldwide prevalence of chronic diseases [1,2]. Considering the importance of maternal nutrition, the Government of India has instituted various initiatives to enhance maternal and child health outcomes, notably the Integrated Child Development Services (ICDS) system. The ICDS program, initiated in 1975, encompasses a network of Anganwadi centers, where Anganwadi workers (AWW) provide a broad range of services, including supplementary nutrition, immunization, health assessments, medical referral services, and nutrition and health education for women [3,4]. The Government of India has also initiated several programs to enhance maternal health, which include POSHAN (PM’s Overreaching Scheme for Holistic Nourishment) Abhiyaan, Pradhan Mantri Matru Vandana Yojana (PMMVY), National Health Mission, Anaemia Mukt Bharat program, National Rural Livelihoods Mission, and Task Force on Healthy and Balanced Diets [5,6].

Notwithstanding comprehensive operations and significant governmental backing, the engagement of pregnant and lactating women with nutrition assistance from Anganwadi Centres remains inconsistent and insufficient in various locations, particularly in rural and underserved communities [7]. The primary focus of Anganwadi personnel is on nutritional supplements, resulting in the neglect of other crucial activities such as counselling of antenatal care, birth preparedness, and recognition of danger signs [8]. In Central India, socioeconomic constraints such as poverty, limited literacy, and prevailing male dominance substantially impede the utilisation of healthcare services. These determinants similarly affect the uptake of maternal health interventions, including nutritional supplementation, underscoring the need to address these systemic barriers to improve maternal outcomes. The purpose of this research is to determine the extent of Anganwadi service utilisation among pregnant women. The findings may also help assess the quality of services provided by Anganwadi centres and highlight the need to address existing barriers and implement appropriate measures to improve utilisation rates.

## Materials and methods

Aims and objectives

The aims and objectives of this study are: (1) To determine the level of utilisation of Anganwadi services by pregnant and lactating mothers in the study area, and (2) To identify the barriers affecting the utilisation of Anganwadi services by pregnant and lactating mothers at the study site.

Materials and methods

This was a cross-sectional observational study conducted at a Rural Health Training Center (RHTC) in Central India over a duration of three months (November 2023 to January 2024). Inclusion criteria consisted of pregnant women and lactating mothers present during the data collection period and willing to participate. Exclusion criteria included women who were unwilling to participate, not available during the research team's visit or had a serious illness preventing them from completing the interview.

Sample Size

The sample size required for the study was calculated using the prevalence of utilization of ICDS among pregnant women in urban areas (primary outcome), which was considered 38.8% from the National Family Health Survey (NFHS-4) (2015-16); with 5% absolute precision and 10% non-response rate, the final sample size of 365 was calculated.

Participants were enrolled by convenience sampling technique. They were interviewed using a pre-designed and face-validated questionnaire [8]. The questionnaire encompassed demographic characteristics, awareness of Anganwadi services, and factors influencing utilization, including reasons for non-utilization of supplementary nutrition. Participants provided written informed consent, ensuring confidentiality and emphasizing voluntary participation.

Statistical Analysis

Descriptive statistics summarized the data, while chi-squared test was used to see the association between sociodemographic variables and utilization of Anganwadi services and to explore reasons for non-utilization of Anganwadi services. SPSS version 26 (IBM Corp., Armonk, NY, USA) was used for statistical analysis, with significance set at p < 0.05.

## Results

**Figure 1:**
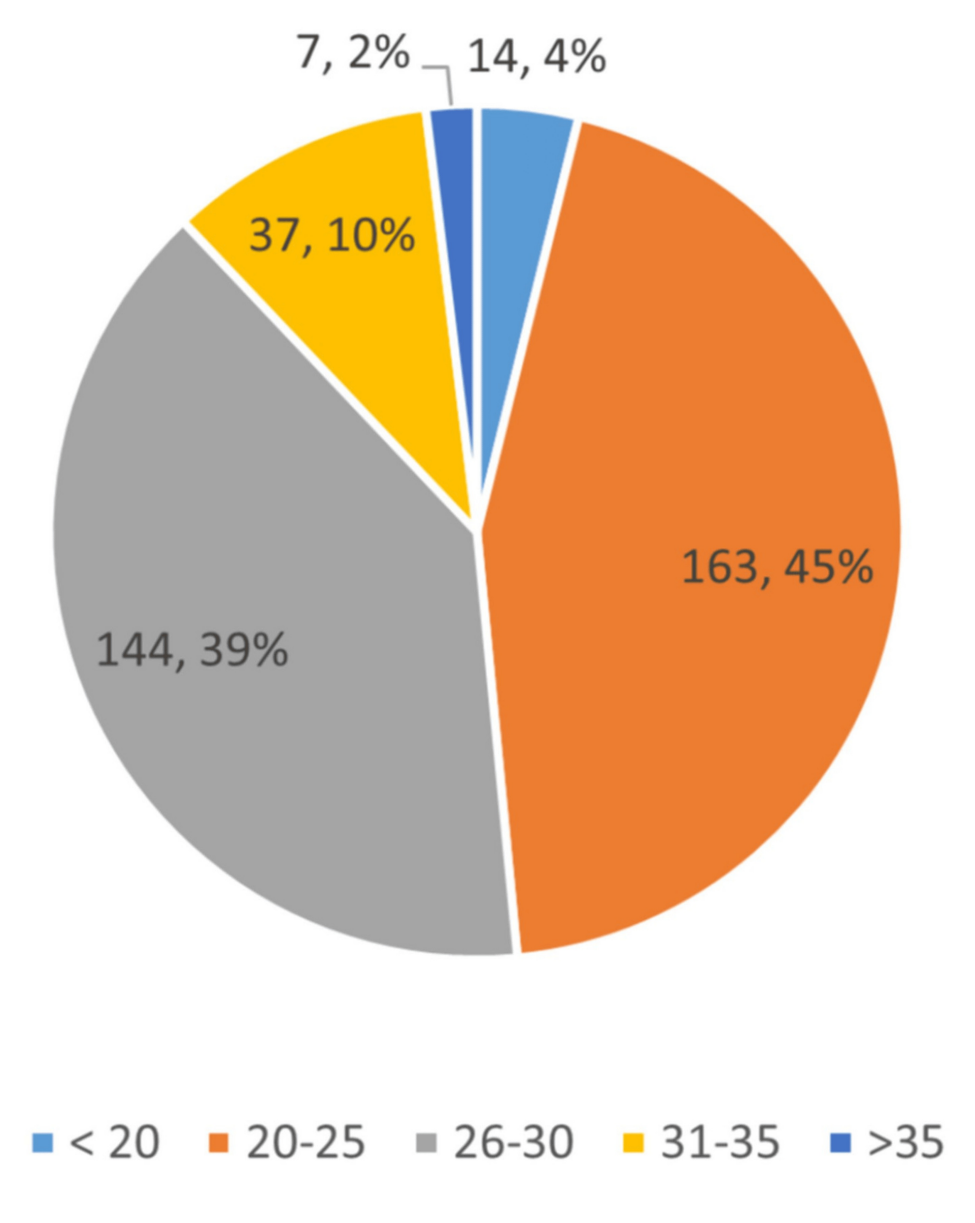
Age Distribution

The study enrolled a total of 365 participants. Mean age of study participants was 26.27 years with a standard deviation of 3.726. Regarding age distribution, the majority of participants fell within the age range of 20 to 30 years, with 45% aged between 20 and 25 years and 39% aged between 26 and 30 years (Figure 1).

All participants in the study were found to reside in rural areas, indicating a significant rural representation. A substantial number of women were found to be graduates (35.8%), indicating a relatively high level of tertiary education attainment. About 61.91% of the husbands of all research participants had less education than a bachelor's degree when their qualifications were assessed. The study participants' family structure was analysed, revealing a predominant prevalence of joint families and majority of participants were classified under the category of middle class (86.02%). The occupation distribution among the study participants showcased a predominant representation of homemakers (79.1%) (Table 1).

**Table 1:** Sociodemographic Details

Variables		N = 365 (Total Study Population)
Age (mean+/-SD)		26.27 (+/-3.73)
Pregnant women		209 (57%)
Lactating women		156 (42%)
Location (Rural)		365 (100%)
Education level of women	Illiterate	02 (0.54%)
	Primary School	01 (0.27%)
	High School	92 (25.2%)
	High Secondary	117 (32.05%)
	Graduate	131 (35.8%)
	Postgraduate	22 (6.02%)
Husband Education	less than Graduation	226 (61.91%)
	Graduate and above	139 (38.08%)
Type of family	Joint Family	252 (69%)
	Nuclear Family	113 (31%)
Socioeconomic status	Upper Class	30 (8.2%)
	Upper Middle	102 (27.9%)
	Lower Middle	212 (58%)
	Upper Lower	21 (5.7%)
	Low	0
Working status of participants	Homemaker	289 (79.17%)
	Working women	76 (20.82%)

The study provides insights into the awareness level among study participants regarding the Anganwadi Centres in Central India. Majority of participants (91%) were aware of the location of Anganwadi Centres in their area. About 72% of the study participants reported receiving MCP (Mother and Child Protection) cards from Anganwadi centres. About 91% of participants were aware of the availability of supplementary nutrition at Anganwadi centres, 83% were aware of the provision of iron-folic acid (IFA) and calcium supplements and 63% of the study participants reported receiving supplementary nutrition from the center (Table 2).

**Table 2:** Anganwadi services awareness and benefits IFA: Iron-folic acid; MCP: Mother and Child Protection

		N = 365 (Total Study Population)
Awareness of Anganwadi centre location	Yes	333 (91%)
	No	32 (9%)
Awareness of Services Available at Anganwadi Centres	Supplementary Nutrition	332 (91%)
	Heath Education	333 (91%)
	Health Checkup	330 (90%)
	Provision of IFA	303 (83%)
	Provision of Calcium	303 (83%)
	Deworming	106 (29%)
Issuance of MCP Cards by Anganwadi centres to study participants	Yes	263 (72%)
	No	58 (16%)
	Not applicable	44 (12%)
Receipt of Supplementary Nutrition Among Study Participants at Anganwadi Centres	Yes	230 (63%)
	No	135 (37%)

**Figure 2:**
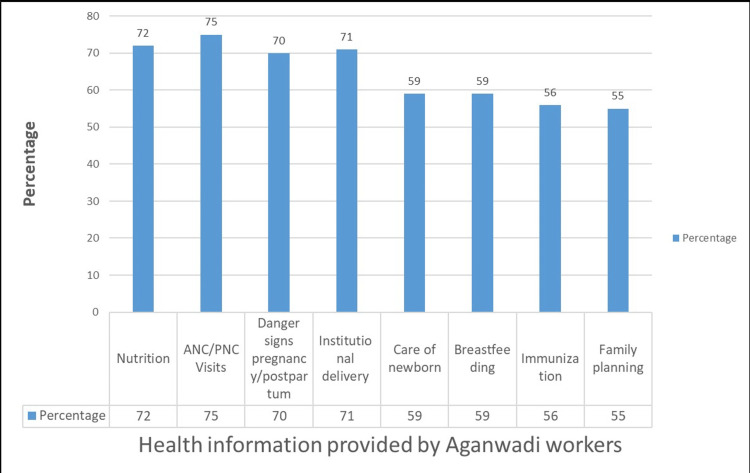
Receipt of Health Education from Anganwadi

The study highlighted the significant role of Anganwadi teachers in disseminating crucial health information related to maternal and child healthcare, covering various aspects from pregnancy to postpartum care and child-rearing practices (Figure 2).

**Figure 3:**
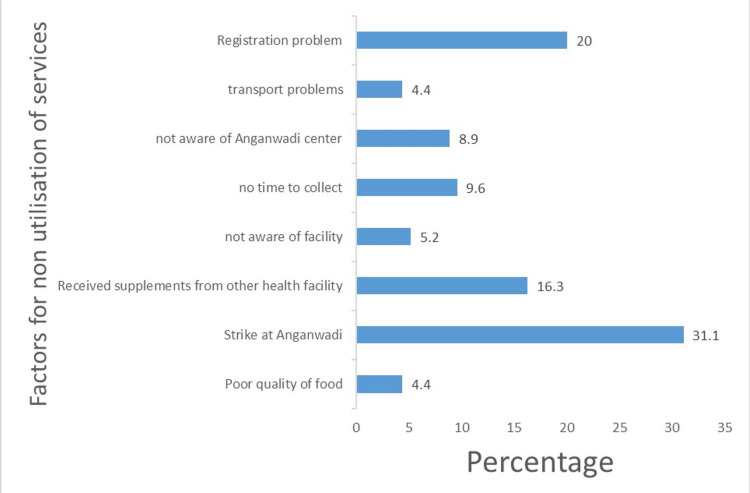
Reasons of not utilising supplementary nutrition among beneficiaries

The study delineates the reasons behind the non-utilization of supplementary nutrition among pregnant and lactating women accessing Anganwadi services in Central India. Notably, the most prevalent reason cited by 31.1% of participants was the occurrence of strikes at Anganwadi centres indicating disruptions in service delivery. Additionally, registration issues emerged as a significant barrier, with 20% of participants facing obstacles in accessing supplementary nutrition due to registration hurdles (Figure 3).

Table 3 showed the association between various sociodemographic factors and utilization of Anganwadi services among pregnant women and lactating mothers. There were no significant differences in service utilization based on age or education level of women (p>0.05). However, there was a significant association between the education level of husbands and service utilization, with a higher proportion of utilization among women whose husbands had higher levels of education (p = 0.031). Additionally, socioeconomic status demonstrated a significant association with service utilization (p = 0.0), with higher utilization among women from lower-middle and middle socioeconomic status households compared to those from upper-middle and upper-class households. These findings highlight the need for targeted interventions that involve not only women but also their families and consider socioeconomic factors to improve the utilization of Anganwadi services among pregnant women and lactating mothers in rural areas.

**Table 3:** Impact of sociodemographic factors on health care utilization

Characteristics of beneficiaries		Utilization		Total % (n=321) Registered women	Significance
		Yes	No		
Age of women	<20 years	7 (63.6%)	4 (36.4%)	11	P-value = 0.57
	20-30 years	200 (72.7%)	75 (27.3%)	275	
	>30 years	23 (65.7%)	12 (34.3%)	35	
Education of women	Illiterate	2 (66.7%)	1 (33.3%)	3	P-value = 0.3
	High School	60 (73.2%)	22 (26.8%)	82	
	High Secondary	85 (80.2%)	21 (19.8%)	106	
	Graduation and above	83 (63.8%)	47 (36.2%)	130	
Husband Education	Less than graduation	159 (79.5%)	41 (20.5%)	200	P-value <0.05
	Graduate or above	71 (58.7%)	50 (41.3%)	121	
Working status of women	Homemaker	189 (74.1%)	66 (25.9%)	255	P-value <0.05
	Working	41 (62.12%)	25 (37.87%)	66	
Socioeconomic status	Lower middle	148 (78.7%)	40 (21.3%)	188	P-value <0.05
	Middle	16 (84.2%)	3 (15.8%)	19	
	Upper middle	55 (62.5%)	33 (37.5%)	88	
	Upper class	11 (42.3%)	15 (57.7%)	26	

## Discussion

Anganwadi services in India serve as a cornerstone of the nation's public health infrastructure, offering a comprehensive range of essential services aimed at promoting the health, nutrition, and development of vulnerable populations, especially pregnant and lactating women, and young children. Anganwadi workers, often drawn from the local community, play a crucial role in delivering these services, acting as frontline healthcare providers and community educators [9]. The present study contributes to understanding the utilization patterns and challenges faced by pregnant and lactating women accessing Anganwadi services in Central India.

Our study revealed a notable level of awareness among participants regarding the various services offered at Anganwadi centres, with 91% of participants being knowledgeable about supplementary nutrition, health education, and health checkup services. Similarly, a study conducted in Telangana [3] reported that 86.66% of mothers possessed average knowledge regarding Anganwadi services. Additionally, research by Jose et al. indicated a similar awareness level, with 84.5% of pregnant women being aware of the Anganwadi centre [8]. This high level of awareness among study participants regarding the location, beneficiaries, and services provided at Anganwadi centres suggests the effectiveness of existing outreach efforts and communication strategies.

Understanding the educational and occupational profiles of participants and their partners is essential for creating programs and services that address the unique requirements and obstacles faced by women, as the majority of Indian women rely on their husbands for even the smallest decisions. Women may be more open to using healthcare services and have greater access to information about them if their spouses have higher education levels.

In our study, 63% of participants reported receiving supplementary nutrition from Anganwadi centres, indicating significant access to essential nutritional support for pregnant and lactating women. In contrast, another study reported that only 48% of participants received supplementary nutrition from Anganwadi centres [8]. However, in the study by Paul et al. [6], 57.8% beneficiaries utilized nutritional support. These findings underscore the importance of ensuring widespread access to nutritional support for pregnant and lactating women to improve maternal and child health outcomes.

On comparing the different factors influencing healthcare utilization among women in different settings, our study aligns with research conducted in the urban colony of Delhi [6] and one conducted in Ernakulam District, Kerala [10], which identified lack of time due to work as a significant barrier to the utilization of services. Similarly, the study in Telangana [3] and a qualitative study by Mishra et al. [11] emphasize the importance of factors such as infrastructure facilities, logistic support, quality of health facility, and community participation in improving the efficiency of Anganwadi centres, which indirectly impacts the motivation of beneficiaries to access services.

Sociodemographic factors, including age, education, occupation, and socioeconomic status, were found to be significant determinants of healthcare utilization among the study participants. Higher levels of education among both women and their husbands were associated with increased healthcare utilization, indicating the importance of educational attainment in fostering health-seeking behaviors. However, as per the report of NFHS-5 survey, socioeconomic status had an inverse relation with Anganwadi service utilisation [12].

An overview of the reasons for the non-utilization of supplementary nutrition services among pregnant and lactating women highlighted various barriers hindering the uptake of this vital service. Notably, the most prevalent reason (31.1%) was the occurrence of strikes at Anganwadi centres indicating disruptions in service delivery. Therefore, it is essential to consider such administrative factors affecting service providers when aiming to improve overall service utilisation. Additionally, registration issues emerged as a significant barrier, with 20% of participants facing obstacles in accessing supplementary nutrition. Logistic challenges such as the distant location of Anganwadi centres (4.4%) and lack of awareness about their location (8.9%) were cited by a subset of participants. Furthermore, constraints on time (9.6%) and limited awareness about the availability of supplementary nutrition services (5.2%) were identified as additional barriers. Interestingly, 16.3% of participants reported receiving supplementary nutrition through other schemes of the centre, suggesting potential duplication of services or confusion regarding eligibility criteria. Moreover, concerns about the poor quality of food grains were cited by a small percentage of participants (4.4%). These findings underscore the multifaceted nature of barriers hindering the utilization of supplementary nutrition services among pregnant and lactating women, highlighting the importance of addressing systemic issues and improving access to ensure the equitable provision of essential nutritional support. Our findings align with studies conducted in rural Telangana [3] and urban resettlement colonies of Delhi [6], rural South Karnataka [8], which identified issues such as poor awareness of services, logistical challenges, and concerns about the quality of supplementary nutrition.

While this study provides important insights into the utilization of Anganwadi services among pregnant and lactating women in Central India, it is not without limitations. First, the cross-sectional design limits the ability to establish causality between associated factors and service utilization. Second, the study was conducted in a single rural health training centre, which may limit the generalizability of the findings to other geographic or urban settings. Third, recall bias may have affected participant responses regarding past utilization or awareness of services. Additionally, convenience sampling may have introduced selection bias, and participants who were not available during the study period were excluded, potentially omitting a subset of the population with differing utilization patterns. Despite these limitations, the study offers valuable insights and serves as a foundation for future, broader investigations.

## Conclusions

This study sheds light on the challenges for utilisation of services such as logistical hurdles and concerns about service quality persist. Sociodemographic factors, particularly the education level of husbands, significantly influence service utilization. The findings have significant implications for policy development and program planning, highlighting the need for targeted interventions to ensure equitable access to maternal and child healthcare services.
